# Supplementary material for: High prevalence of long-term olfactory disorders in healthcare workers after COVID-19: A case-control study
Source: PLoS One. 2024 Jul 1;19(7):e0306290. doi: 10.1371/journal.pone.0306290 (PMC11216562; doi:10.1371/journal.pone.0306290)
Supplement: S1 Fig — Scatterplot illustrating the association between TDI score and (A) days since infection or (B) age of individuals who had had COVID-19 (COVID+, in orange) and COVID-19 naïve individuals (COVID-, in blue). Spearman correlations showed that TDI score was not significantly affected by the number of days since infection (N = 71, r = -.1, p = .43), but significantly declined with age in the COVID+ group (r = -.28, p = .005) but not in the COVID- group (r = -.27, p = .09). (PDF) [file pone.0306290.s001.pdf]

High prevalence of long-term olfactory disorders in healthcare workers after COVID-19: a case-control study

Supplementary Information

S1 Fig

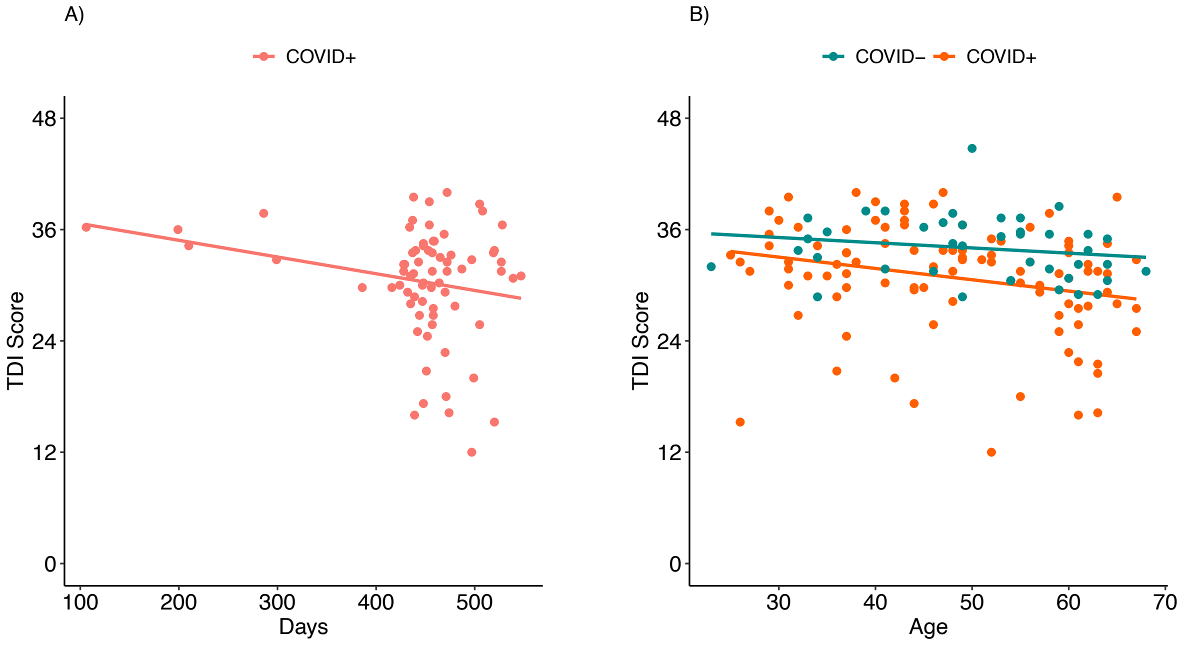

S1 Fig. Scatterplot illustrating the association between TDI score and (A) days since infection or (B) age of individuals who had had COVID-19 (COVID+, in orange) and COVID-19 naïve individuals (COVID-, in blue). Spearman correlations showed that TDI score was not significantly affected by the number of days since infection (N = 71, r = -.1, p = .43), but significantly declined with age in the COVID+ group (r = -.28, p = .005) but not in the COVID- group (r = -.27, p = .09).
